# Single-cell RNA sequencing algorithms underestimate changes in transcriptional noise compared to single-molecule RNA imaging

**DOI:** 10.1016/j.crmeth.2024.100933

**Published:** 2024-12-10

**Authors:** Neha Khetan, Binyamin Zuckerman, Giuliana P. Calia, Xinyue Chen, Ximena Garcia Arceo, Leor S. Weinberger

**Affiliations:** 1 Gladstone|UCSF Center for Cell Circuitry, University of California, San Francisco, San Francisco, CA 94158, USA; 2 Department of Biochemistry and Biophysics, University of California, San Francisco, San Francisco, CA 94158, USA; 3 Department of Pharmaceutical Chemistry, University of California, San Francisco, San Francisco, CA 94158, USA; 4 Institute for Evolvable Medicines, Oakland, CA, USA; 5 Autonomous Therapeutics, Inc., Rockville, MD, USA

**Keywords:** transcriptional noise, noise-enhancer molecule, single-cell RNA sequencing, scRNA-seq, single-molecule RNA FISH, smFISH

## Abstract

Stochastic fluctuations (noise) in transcription generate substantial cell-to-cell variability. However, how best to quantify genome-wide noise remains unclear. Here, we utilize a small-molecule perturbation (5′-iodo-2′-deoxyuridine [IdU]) to amplify noise and assess noise quantification from numerous single-cell RNA sequencing (scRNA-seq) algorithms on human and mouse datasets and then compare it to noise quantification from single-molecule RNA fluorescence *in situ* hybridization (smFISH) for a panel of representative genes. We find that various scRNA-seq analyses report amplified noise—without altered mean expression levels—for ∼90% of genes and that smFISH analysis verifies noise amplification for the vast majority of tested genes. Collectively, the analyses suggest that most scRNA-seq algorithms (including a simple normalization approach) are appropriate for quantifying noise, although all algorithms appear to systematically underestimate noise changes compared to smFISH. For practical purposes, this analysis further argues that IdU noise enhancement is globally penetrant—i.e., homeostatically increasing noise without altering mean expression levels—and could enable investigations of the physiological impacts of transcriptional noise.

## Highlights

• scRNA-seq validates that IdU orthogonally amplifies transcriptome-wide noise
• smFISH validates IdU-induced noise amplification for most genes
• scRNA-seq algorithms underestimate the fold change in noise compared to smFISH
• The underestimation of changes in noise holds even after extrinsic factor corrections

## Motivation

Cell-to-cell variability in isogenic populations is predominantly attributed to the stochastic fluctuations (i.e., noise) in transcription. However, the quantification of this noise, particularly on a genome-wide scale, remains an open question. To address this general question, we utilize a small-molecule perturbation reported to amplify transcriptional noise and assess numerous single-cell RNA sequencing (scRNA-seq) algorithms for their ability to quantify noise amplification while also comparing the results to noise quantification from single-molecule RNA fluorescence *in situ* hybridization (smFISH) imaging. In this way, we seek to address whether 5′-iodo-2′-deoxyuridine (IdU) amplifies noise in a globally or partially penetrant manner and what scRNA-seq algorithm is most appropriate for quantifying transcriptional noise.

## Graphical abstract

**Figure:**
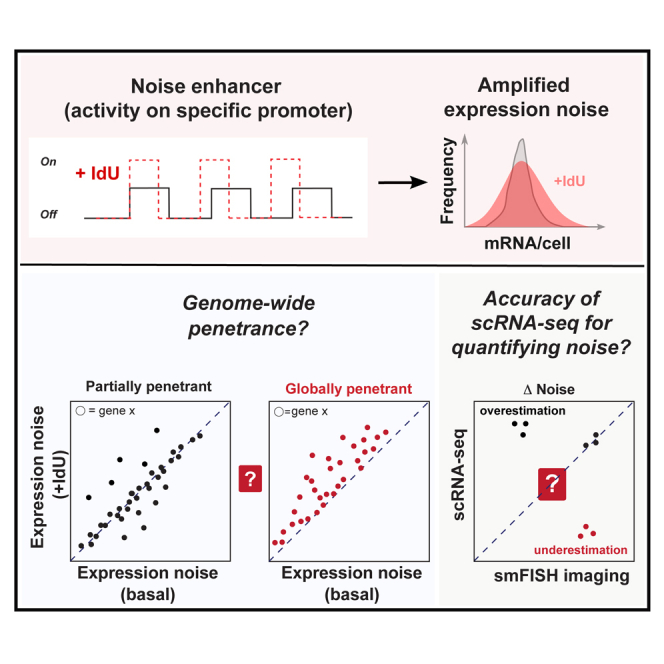

## Teaser

Khetan et al. analyze changes in transcriptional noise after perturbation with a molecule (IdU) that orthogonally amplifies noise (i.e., without altering mean expression). They find that IdU amplifies noise in a globally penetrant manner, but commonly used scRNA-seq algorithms underestimate the fold change in noise amplification compared to single-molecule FISH (smFISH) imaging.

## Introduction

Cell-to-cell variability is an unavoidable consequence of the biochemical processes occurring in individual cells^1^ and has been implicated in cell-fate specification decisions ranging from HIV latency to cancer.^2^^,^^3^^,^^4^ While a portion of cell-to-cell variability arises from extrinsic factors (e.g., cell size, cycle phase, or microenvironment), a substantial body of literature has demonstrated that, in isogenic populations of cells—particularly mammalian cells—a large fraction of the variability originates from intrinsic sources, such as stochastic fluctuations (noise) in transcription.^5^^,^^6^ These intrinsic stochastic fluctuations can be quantitatively accounted by gene expression “toggling” between active and inactive states, which produces episodic “bursts” of transcription, and a theoretical formalism commonly known as the two-state or random-telegraph model of gene expression is often used to fit these expression bursts.^7^^,^^8^^,^^9^^,^^10^ Notably, transcriptional bursts are known to transmit to the protein level, and expression noise is often amplified by nuclear export and mRNA processing in the cytoplasm.^11^^,^^12^ Ultimately, these transcriptional bursts generate a substantial fraction of the measured noise^13^^,^^14^ and influence cell-fate specification decisions.

Nevertheless, measurements of transcriptional noise, particularly on a genome-wide scale, face several technical challenges. Specifically, how best to quantify noise across the transcriptome remains an open question, in part because there are no established methods to perturb noise across the transcriptome, which would be critical to benchmark noise measurement approaches. Unfortunately, perturbing expression noise without altering the mean expression level (i.e., orthogonal perturbation) has been challenging since, for most physical processes, the noise and mean levels are inherently linked. Intuitively, the reason for this linkage is that cellular processes, at their most fundamental level, are molecular birth-death processes, and such birth-death processes are Poissonian (or often super-Poissonian), where the variance (σ^2^) necessarily equals the mean (μ) (i.e., as μ changes, so does σ^2^)—notably, the two-state model is an extension of the simple birth-death processes and generates super-Poisson distributions,^11^^,^^12^^,^^14^ where the mean and noise remain tightly correlated but the variance is always greater than the mean (σ^2^ > μ). Ultimately, the practical outcome of this intrinsic linkage is that the common metric for quantifying expression noise, the coefficient of variation (CV; which is the standard deviation normalized by the mean: σ/μ), necessarily decreases as the mean increases (CV ∝ 1/μ).^15^ Consequently, in some cases, the normalized variance, or the Fano factor (σ^2^/μ), can be used to compare noise for processes with different mean values, as the Fano factor does not scale with the mean.^16^^,^^17^ Regardless, perturbations that orthogonally modulate noise without changing the mean remain a challenge.

Historically, a mechanism for breaking the 1/μ dependence of CV and orthogonally modulating noise is via specific autoregulatory architectures (e.g., feedback and feedforward).^12^^,^^18^ However, more recently, small molecules called “noise enhancers” were found to generate increased expression noise without altering the mean expression level^19^ by a process known as homeostatic noise amplification^20^—a notable contrast to transcriptional activators, which increase mean expression levels. We reported the molecular mechanism for one particular class of noise-enhancer molecule, pyrimidine-base analogs such as 5′-iodo-2′-deoxyuridine (IdU), and used single-cell RNA sequencing (scRNA-seq) to show that IdU increases noise across the transcriptome. Unfortunately, scRNA-seq, despite its utility in measuring genome-wide expression, suffers from well-established issues of technical noise^21^^,^^22^ due to small inputs of RNA, varying sequencing depth, amplification bias, dropouts, and differences in capture ability^21^^,^^22^ that could obscure the quantification of IdU penetrance and IdU-mediated degrees of noise enhancement.

Several algorithms and analysis pipelines have been developed to address the challenges of scRNA-seq analysis.^23^^,^^24^^,^^25^ These algorithms include approaches to minimize biases from data transformation, varying sequencing depth and dropouts and incorporating methods to accurately delineate between technical and biological noise. However, in general, there is no consensus on the appropriate pipeline to quantify transcriptional noise, and the choice of algorithm often depends on the biological system being studied and the specific scientific question being addressed.

Here, we set out to determine which scRNA-seq pipelines were appropriate for quantifying transcriptional noise using the noise-enhancer molecule IdU^20^ as a noise perturbation. Specifically, we also set out to determine if IdU acted as a globally or partially penetrant noise-enhancer molecule. We examined multiple scRNA-seq algorithms using a mouse embryonic stem cell (mESC) dataset with and without IdU treatment, where each algorithm can account for experimental and sampling biases and is intended to minimize extrinsic and technical noise.^26^ Each algorithm identified a different proportion of the genes exhibiting amplified noise as well as differences in the magnitude of noise amplification. A follow-up scRNA-seq profiling of human Jurkat T lymphocytes confirmed that IdU-mediated noise amplification is not restricted to the specific biology of mESCs. To validate scRNA-seq measurements, we then employed single-molecule RNA fluorescence *in situ* hybridization (smFISH)—the gold standard for mRNA quantification due to its high sensitivity for mRNA detection^27^—to probe a panel of genes from across the transcriptome that span a wide array of expression levels and represent a range of cellular functions. Collectively, these analyses indicate that IdU amplifies the noise of most genes (globally penetrant) and could, in principle, be a candidate to probe the physiological roles of expression noise for diverse genes of interest.

## Results

### Alternate scRNA-seq algorithms generate differing profiles of expression noise, indicating noise amplification (ΔFano > 1) for ∼90% of expressed genes

**Figure 1:**
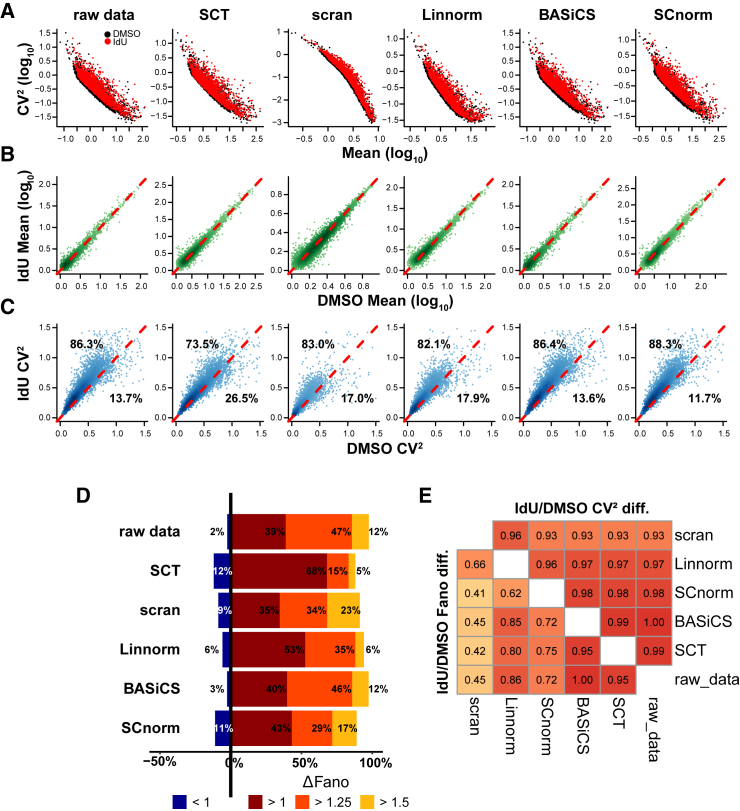
Common scRNA-seq normalization algorithms generate different quantifications of mRNA noise (A) scRNA-seq analysis of CV^2^-versus-mean for 4,456 transcripts in mESCs treated with IdU (red) or DMSO control (black) as analyzed by commonly used normalization algorithms: SCTransform (“SCT”), scran, Linnorm, BASiCS, or SCnorm. (B) Mean expression for each of the 4,456 transcripts in the presence and absence of IdU using each normalization algorithm; none of the normalizations algorithms generate substantial changes in mean expression for IdU-treated cells. (C) CV^2^ for each transcript in the presence and absence of IdU using each normalization algorithm; different algorithms generate substantially different fractions of transcripts with amplified noise, ranging from ∼70% of transcripts with amplified noise (SCT) to ∼88% of transcripts with amplified noise (SCnorm). (D) Quantification showing percentages of transcripts with indicated Fano factor fold changes between IdU-treated and control cells. (E) Pearson correlation coefficients between fold changes in noise metrics between IdU-treated and control cells. Shown are correlations between the indicated methods quantifying fold changes in CV^2^ (top right heatmap) and Fano (bottom left heatmap). See also Figure S1.

To examine how different scRNA-seq normalization methods influence the quantification of IdU-mediated noise amplification, we employed commonly used algorithms to analyze scRNA-seq data from IdU-treated versus DMSO-treated (control) mESCs.^20^ This high-quality dataset consists of a few hundred deeply sequenced cells (>60% sequencing saturation) and allows reliable noise quantification even for moderately expressed genes. Yet, this experimental design is prone to technical noise, stemming from varying sequencing depths and the absence of biological replicates, which could compromise the evaluation of the actual IdU-mediated transcriptional noise amplification. To obtain a more rigorous measurement of noise enhancement penetrance, we compared noise quantification from a simple normalization approach, i.e., normalized by sequencing depth (described in STAR Methods, and referred to here as a raw method) to the five established scRNA-seq algorithms (Figure 1): SCTransform,^28^ scran,^29^ Linnorm,^30^ BASiCS,^31^^,^^32^ and SCnorm.^33^ SCTransform is a commonly used normalization method that employs a negative binomial model, including regularization and variance stabilization steps, while *scran* estimates cell-specific size factors for normalization by deconvolving pooled expression data from groups of cells. Linnorm utilizes homogenously expressed genes to estimate factors for transformation followed by variance stabilization. SCnorm groups genes based on count-depth relationships and uses quantile regression to generate normalization factors, while BASiCS employs a hierarchical Bayesian framework to simultaneously estimate model parameters for normalization factors, technical noise, and both Poissonian and super-Poissonian noise explicitly.

Despite their substantially different technical schemes, analysis with each of these normalizations indicated that IdU induces a substantial amplification of noise (CV^2^) for most expressed genes (Figure 1A; Wilcoxon rank-sum test for CV^2^: *p* < 10^−17^ for all methods). The IdU-induced noise amplification appeared to be homeostatic (Figure 1B), with mean expression levels largely unchanged by IdU under all algorithms (Wilcoxon rank-sum test for mean values: *p* > 0.02 for SCnorm and scran and *p* > 0.1 for SCTransform, Linnorm, and BASiCS). However, each algorithm calculated a somewhat different percentage of expressed genes with increased CV^2^, ranging from 73% to 88% of genes exhibiting increased noise (Figure 1C). Quantification of noise by analysis of the normalized variance (i.e., Fano factor), which, in principle, eliminates the scaling dependence on mean expression level, showed a similar profile (Figure 1D; Wilcoxon rank-sum test for Fano: *p* < 10^−70^ for all methods). While every algorithm showed that a majority of expressed genes exhibit amplified noise by the Fano factor, each calculated a different penetrance (i.e., percentage of noise-amplified genes), and there were substantial differences in the magnitude of noise amplification among those transcripts with amplified noise.

Notably, the analysis further confirmed that BASiCS yielded minimal data transformation when compared to the other algorithms (Figures S1 and S2). As a negative control, the DMSO-treated samples were randomly split into two subpopulations and the noise metrics of both groups compared, and no significant global change in CV^2^ or Fano factor between the two randomly split groups was found (Figures S2A and S2B).

To explore the effect of IdU on transcriptional noise in a different biological context and under a different experimental setup, a separate scRNA-seq profiling ±IdU was performed on a second cell line: human Jurkat T lymphocytes. The Jurkat cell line (a suspension cell line) is more homogeneous than adherent stem cells^11^ and exhibits a cell cycle time of about 24 h,^34^ which is 2–3× longer than mESCs.^35^ IdU treatment duration and concentration were modified for Jurkat cells to account for the longer cell-doubling time and altered sensitivity of Jurkat cells to IdU compared to mESCs, as determined by viability analysis under varying IdU doses (data not shown). Two biological replicates of Jurkat cells, each treated with DMSO or 20 μM IdU for 48 h, were profiled by moderate-depth scRNA-seq, and a comparison between the replicates confirmed that noise quantification by all algorithms is highly reproducible (Figure S2C) and that the batch-effect contribution to noise is negligible (Figure S2D).

In this Jurkat scRNA-seq dataset, sequencing depth bias was minimized and largely eliminated, as all samples and replicates exhibited highly uniform coverage as estimated by unique molecular identifier (UMI) counts per sample (Figure S2E). Analysis with the different normalization methods indicated a consistent increase in noise metrics despite a lower fraction of affected genes compared to mESCs (Figure S3). Overall, these results confirm that IdU enhances noise in diverse human and mouse cell types and that the extent of noise generated depends (at least partly) on the cell division rates (though other possible mechanisms may account for this difference; see discussion).

### RNA quantification by smFISH indicates widespread penetrance of IdU-induced noise amplification

**Figure 2:**
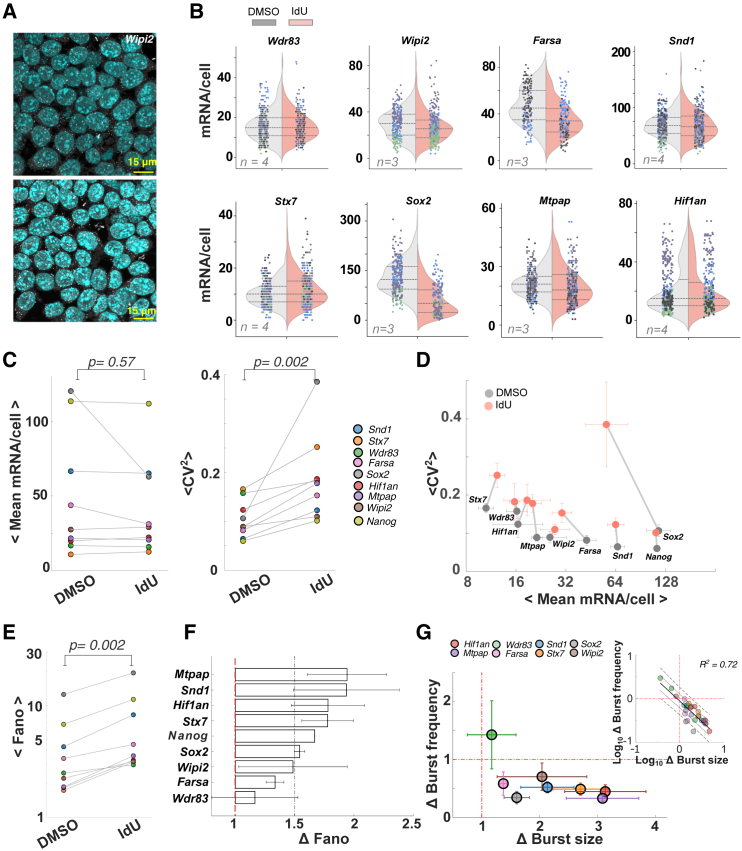
smFISH verification of noise amplification (A) Representative smFISH images of *Wipi2* transcripts (white dots) in mESCs treated with DMSO (control, top) or IdU (treated, bottom), with DAPI-stained nuclei (blue). Scale bar: 15 μm. See also Figure S4D. (B) Distribution of mRNA abundance per cell from all replicates in DMSO- (gray) and IdU (red)-treated samples. Colors in scatter correspond to replicates (*n*: number of replicates). Dashed lines represent median and first and third quartiles of mRNA distribution. (C) IdU-induced changes in gene expression quantified as mean mRNA/cell (μ) and increase in noise as mean CV^2^ (σ^2^/μ^2^) suggest statistical insignificance and significance, respectively, using the paired Wilcoxon signed-rank test on the means across replicates. Colors represent genes. (D) CV^2^-versus-mean mRNA abundance per cell for each gene is represented. DMSO (gray circles) and IdU (red circles). Error bars indicate ± SEM. (E) Increase in Fano factor (FF i.e., σ^2^/μ) in IdU-treated cell population is statistically significant from the untreated DMSO populations, inferred from paired, one-sided Wilcoxon signed-rank test. See also Figure S5. (F) IdU/DMSO (i.e., fold change, as Δ) in Fano factor (i.e., FF IdU/FF DMSO) across the genes is plotted. Red lines are a guide for the eyes for the axis of no change. See also Figure S5. (G) The Δ in IdU- to DMSO-treated samples for burst frequency and burst size are plotted, as estimated from the fits to the negative binomial distribution. Error bars correspond to the SEM. Red lines are a guide for eyes for the axis of no change. See also Figure S6. Inset: the log-transformed Δ (i.e., IdU/DMSO) in burst frequency and burst size are plotted for each gene from each replicate. Colors indicate genes. Solid black line indicates the fit to the linear regression with standard error of estimates (dashed and dotted lines for 2 and 1, respectively).

To directly quantify RNA levels in individual cells using a non-sequencing-based approach, we employed smFISH, a well-established imaging method to assess noise and the gold standard for quantitative assessment of mRNA expression.^36^ While smFISH enables the quantification of mRNA abundance in individual cells, it is a relatively low-throughput method requiring distinct fluorescent probes and image quantification for each transcript species of interest. Consequently, to generate a representative view of overall transcription using smFISH, we selected a panel of eight genes (Figures S4A and S4B) that satisfied three criteria: (1) displayed the greatest difference in noise amplification as calculated by the different RNA-seq algorithms (Figure S4B), (2) spanned a wide range of gene expression levels (i.e., across 2 logs) and genome locations (Figure S4A),^37^ and (3) were compatible with an smFISH probe design (i.e., a minimum of 30 probes were predicted to hybridize to the transcript; see Data S3). We also included previously reported smFISH data^20^ from a ninth gene, *Nanog*, to benchmark the smFISH analysis for the effect of IdU-induced amplification of noise. smFISH probe sets for each gene in the panel were generated, cells were imaged in the presence/absence of IdU, and images (Figures 2A and S4C) were segmented and analyzed using FishQuant^38^ to obtain per-cell mRNA counts.

Despite previous observations that IdU-mediated noise amplification is largely independent of cell cycle phase,^20^ we nevertheless accounted for potential contributions from the cell cycle. This analysis is based on an established body of literature that cell size is a surrogate for cell cycle phase,^11^^,^^20^^,^^39^ and we filtered out extrinsic noise by restricting the analysis to cells of similar size (Figure S4D). At least three biological replicates were imaged to quantify per-cell mRNA abundance for each gene and condition, with at least 50 cells used after cell-size-based extrinsic noise filtering (Figure 2B). This analysis revealed significantly greater variance and CV^2^ in mRNA levels in IdU-treated samples compared to controls for most genes, while the change in mean expression was insignificant (Figures 2C, 2D, and S5A). A direct comparison of mRNA CV^2^ values showed significant noise amplification for all the genes in the representative panel without substantial changes in the mean (Figures 2D and S5A), in agreement with the scRNA-seq analysis. Notably, *Sox*2 and *Farsa* appeared to be exceptions, exhibiting decreases in mean expression (see discussion).

To ensure that noise amplification, as reported by CV^2^, could not be explained by changes in mean expression, we also analyzed the Fano factor calculated from the smFISH data (Figures 2E, 2F, and S5B). The absolute Fano factor was >1 for all genes, even in the DMSO-treated controls, indicating the gene expression of the selected genes was inherently bursty (Figure S5B), consistent with previous scRNA-seq and smFISH analyses of mESCs.^20^^,^^40^ Moreover, Fano factor analysis verified that all genes in the panel exhibited significant IdU-induced amplification of noise (Figure 2E). The greatest IdU-mediated noise amplifications (i.e., fold change or Δ in the Fano factor) were for *Mtpap*, *Hif1an*, *Snd1*, *Stx7*, *Nanog*, and *Sox2* (ΔFano factor > 1.5), whereas *Farsa* and *Wdr83* exhibited smaller noise amplifications (Figure 2F).

To address the possibility of over-estimation in the noise metrics due to the variation in cell size, even after filtering for extrinsic noise, we computed corrected noise metrics based on an established linear regression analysis.^39^ This analysis indicates no appreciable differences in the noise estimates (Figures S5C and S5D), confirming a lack of cell-size dependence after extrinsic noise filtering, in contrast to a recent study.^41^

To further verify IdU-mediated noise amplification, we tested if the mechanistic underpinnings^19^ of homeostatic noise amplification were satisfied. Theory predicts that homeostatic noise amplification (i.e., changing the noise without altering the mean level) requires reciprocal changes in at least two transcriptional bursting parameters,^19^ e.g., a decrease in transcriptional burst frequency coupled with a corresponding but reciprocal increase in transcriptional burst size such that the mean number of transcripts remains unchanged. Specifically, the two-state random telegraph model could fit mESC smFISH data, but to account for IdU-mediated noise amplification, the inclusion of an additional “off” state in the model coupled with a feedforward gain was required.^20^ Here, based on established literature,^6^^,^^40^ we first inferred the effective burst size and frequency by fitting the mRNA distributions to a negative binomial distribution (Figures 2G, S6A, and S6C) and then quantified the relative IdU-mediated change in noise—importantly, the negative binomial fitting approach does not explicitly account for underlying molecular mechanisms such as the additional off state or feedforward gain. The fitting further shows a reciprocal relationship between the fold change in burst size and frequency (Figure 2G) in agreement with the model of homeostatic noise amplification. The one exception was *Wdr83*, which showed a different pattern of burst size and frequency and is discussed below.

Overall, these smFISH data further validate the scRNA-seq analysis of transcriptional noise and support that IdU generates a highly penetrant homeostatic amplification of transcriptional noise for 8 out of 9 genes in this panel of genes selected from diverse expression profiles and locations in the genome.

## Discussion

This study set out to determine (1) generally which scRNA-seq algorithm was most appropriate for quantifying transcriptional noise using the noise-amplifying molecule IdU^20^ as a perturbation and (2) specifically if the IdU noise-enhancer molecule acts in a globally penetrant manner.

**Figure 3:**
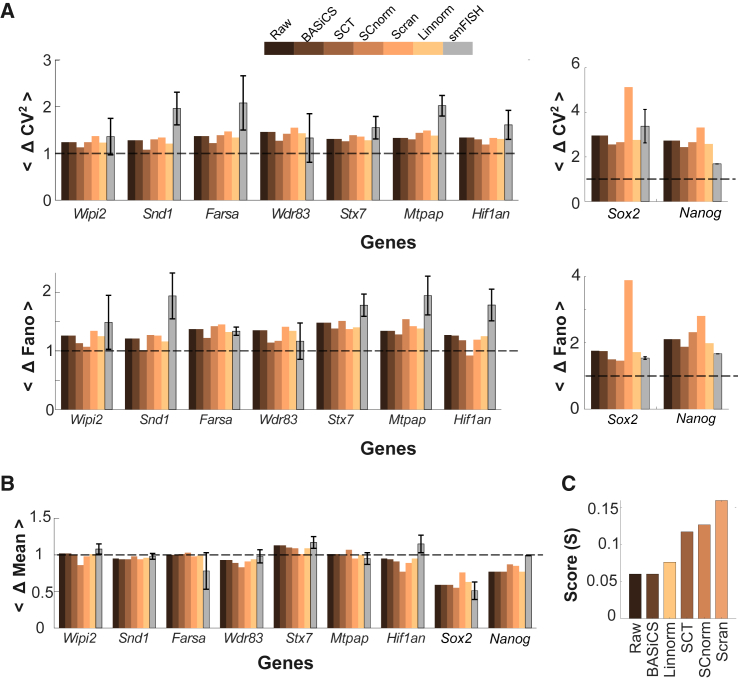
Comparison of noise amplification between smFISH and scRNA-seq (A and B) The fold change (Δ) in (A) noise metrics and (B) mean obtained from different scRNA-seq algorithms is compared to the estimates from measured smFISH across the panel of selected genes. (C) Bar plot depicts the score (*S*), comparing the performance of each scRNA-seq method to the FISH based on the deviation for the three metrics (described in the STAR Methods section).

Analysis of scRNA-seq data using different algorithms indicated that the amplification of noise by IdU is globally penetrant and likely not an artifact of a particular scRNA-seq analysis algorithm. However, the scRNA-seq analysis also indicated that scRNA-seq algorithms generate variable genome-wide noise profiles (Figure 1), with each algorithm suggesting a quantitatively different percentage of penetrance. A second scRNA-seq analysis in Jurkat cells (Figure S3) was consistent with IdU acting as a global noise enhancer, independent of the specific biological context. To validate the scRNA-seq noise analysis, we next used smFISH to examine a panel of genes that exhibited high sensitivity to individual scRNA-seq algorithms and represented both high- and low-expressing genes. smFISH analysis revealed that IdU increased transcriptional noise for 8 out of 9 genes (Figure 2). Overall, the results indicate that most published scRNA-seq algorithms were fairly accurate for the analysis of gene expression noise compared to the smFISH direct measurement (Figures 3A and 3B). Notably, the estimates from the raw method are similar to those from the other algorithms, although all the methods tend to systematically underestimate the fold change in noise metrics compared to smFISH.

While we do not fully understand why scRNA-seq algorithms appear to underestimate the amplification in transcriptional noise compared to smFISH, it seems unlikely that the underestimation is simply due to a normalization factor (i.e., a scalar value) given that this study compares *changes* in noise between the two approaches. It is possible that non-linear data transformations could account for the differences between scRNA-seq and smFISH, but such transformations would likely make 1:1 comparison in the fold change nontrivial. Further, we note that smFISH measures mRNA transcripts directly, while we cannot eliminate potential confounding differences due to technical and methodological factors in scRNA-seq—i.e., UMI counts/cell, a proxy for mRNA abundance in scRNA-seq, could generate confounding effects independently for the estimated mRNA abundance in control versus treated cases. Specifically, scRNA-seq data and smFISH data are on substantially different scales, where smFISH values are discrete counts, while scRNA-seq values are continuous relative counts with values that are orders of magnitude lower than smFISH. We also note that our smFISH analysis rigorously accounts for extrinsic noise factors, while not all of the scRNA-seq algorithms do so, indicating that the underestimation reported is likely a lower bound. Future studies will focus on determining the underlying reasons for this underestimation of changes in noise by scRNA-seq compared to smFISH.

However, a combined score based on the minimal deviation from the smFISH estimates suggest that raw and BASiCS perform relatively better among all the other algorithms tested (Figure 3C). In BASiCS, the fold change in mean, CV^2^, and Fano were directly estimated from normalized counts (using the normalization parameter obtained from the Markov chain Monte Carlo [MCMC] fits) and resulted in minimal data transformation (Figure S1). Additionally, unlike the normalizations in Figure 1, MCMC fits estimate model parameters, including normalization parameters, without transforming the counts and simultaneously provide robust estimates for all the model parameters, including overdispersion—a gene-specific parameter for biological noise that describes non-Poissonian noise after accounting for technical noise and is analogous to CV^2^— which indicates that ∼84% of the genes exhibit a fold increase in biological noise upon IdU perturbation and is comparable to the direct CV^2^ analysis (Figure 1A) which showed ∼86% of genes increased noise. Further, the variance decomposition indicates that ∼26% and ∼75% of the total genes show IdU-induced amplification for Poissonian and super-Poissonian components, respectively, while only ∼3% exhibited an increase in both. Overall, this study suggests that direct estimates of noise, along with BASiCS metrics such as overdispersion, residual variability, and variance decomposition, provide an accurate quantification of transcriptional noise and insights into the phenomenology underlying the gene expression variability. Future studies will focus on determining the metrics that best capture and provide 1:1 comparison between the different absolute mRNA quantification techniques and estimation of the transcriptional noise in physiological contexts.

The Jurkat scRNA-seq analysis of noise (Figure S3D) is generally consistent with previous smFISH analyses in Jurkat cells indicating that 75% of genetic loci have a Fano factor > 1^11^, and the two scRNA-seq biological replicates of the Jurkat experiment confirm that the noise difference between IdU and DMSO samples cannot be attributed to technical bias of the single mESC DMSO and IdU samples alone. However, Jurkat cells appear to exhibit a significantly lower extent of noise amplification. Several mechanisms may account for the observed difference. First, slowly dividing cells, such as Jurkat cells, have slower DNA replication rates; thus, IdU incorporation has less of an impact on transcriptional dynamics due to the relatively fast activity of the base excision repair (BER) pathway, through which IdU is removed from the genome. Second, a technical difference between the DMSO- and IdU-treated mESCs is sequencing depth—while the IdU-treated mESCs were sequenced more deeply, they exhibit lower median UMI counts per cell compared to the DMSO-treated cells (Figure S3E), suggesting a possible reduction in cellular total RNA content upon IdU treatment. This reduction suggests the possibility of lower absolute mean RNA levels masked by normalization methods and higher noise stemming from Poisson scaling, which inflates the noise enhancement effect in the mESC dataset. Third, another technical limitation of our Jurkat scRNA-seq data is the moderate sequencing depth (Figure S3E), allowing noise quantification in only ∼1,000 genes compared to the ∼4,000 genes in mESCs. These ∼1,000 genes comprise the highest expression percentile of all expressed genes. It is possible that noise amplification is less efficient for these very abundant transcripts due to higher RNA stability and/or more continuous than bursty transcription.^42^ Finally, there is also a possibility that noise amplification in mESCs could potentially propagate into cell-state alterations in mESCs, which may introduce transcriptional heterogeneity in these pluripotent stem cells and could reflect potential biological changes in some cells^43^ (a possibility that may be less likely to occur in terminally differentiated Jurkat cells). However, we feel this possibility is unlikely since these biological changes in mESCs were not previously observed^20^ and would likely be reflected by changes in mean expression levels, which were not observed here or previously.^20^

The smFISH replicates, performed on two different microscopes each using different confocal technologies (i.e., spinning disk versus laser scanning), generated similar estimates in the fold change of noise across the panel of genes, which indicates the robustness and lack of bias that emerge from the acquisition differences. The smFISH analysis also allowed the calculation of fold change in burst size and burst frequency for selected genes (Figure 2), and the results were qualitatively comparable to the estimates from our scRNA-seq in an independent recent study using mechanistic models for estimation of the bursting kinetics,^44^ with a quantitative match for the intermediate-abundance gene Mtpap (i.e., log_2_ fold change in burst size and frequency in smFISH: 1.5 and −1.6, while the estimates from the Monod pipeline are 1.3 and −1.5, respectively). The smFISH data verify that IdU amplifies intrinsic transcriptional noise homeostatically (i.e., without altering the mean expression level) for most genes irrespective of genomic location and expression level.

One technical limitation of this study is that smFISH analysis is necessarily low throughput and limited to the subset of genes for which good probe sets can be designed, which limits the spectrum of measurements and the number of genes that can be analyzed. We attempted to mitigate this by exploring genes from across the expression spectrum (Figures S4A and S4B) and analyzing a substantial number of cells per treatment (i.e., at least ∼50 cells per condition and replicates after filtering extrinsic noise), and the estimates from replicates are consistent with statistics obtained from pooled datasets (Figure S6E). Thus, the data herein indicate that IdU likely acts as a noise-enhancer molecule for a large fraction of genes irrespective of their mean expression level.

However, the degree of noise amplification varies. A higher fold change in Fano is observed for *Mtpap* and *Hif1an* genes, which are associated with stress and metabolic processes, and *Syndapin* and *Syntaxin7*, involved in endocytosis and vesicular trafficking, respectively. The pluripotency-associated transcription factors, such as *Sox2* and *Nanog*, exhibit intermediate-noise amplification, while the genes associated with translation and autophagy, i.e., *Farsa* and *Wipi2*, and *Wdr83*, which is involved in mRNA processing and functions as a molecular scaffold, exhibit lower-noise amplification. Interestingly, the magnitude of protein noise in yeast cells is higher for the stress- and metabolism-associated proteins and lower for those involved in protein complexes and translation machinery,^13^^,^^14^ suggesting a potential role of IdU perturbation in investigating the contribution of transcriptional and translational noise. Notably, *Wdr83* is an outlier for its change in noise (Figure 2F), and the estimates of the change in burst size and frequency do fall outside the reciprocal change expected for orthogonal noise amplification (Figure 2G). Considering that IdU increases noise via BER, a genome-wide surveillance pathway, it may be surprising that any gene fails to exhibit an increase in noise. It is possible that the low-throughput nature of smFISH may have obscured IdU-induced noise amplification, or the variation could be inherent to the gene. When replicates for each gene were pooled, the homeostatic increase in noise was maintained (i.e., increase in both the Fano factor and CV^2^ without a significant change in mean) for all genes except *Wdr83*, and the reciprocal relationship between burst size and frequency was also maintained except for *Wdr83* (Figure S6E). However, it should be noted that these population statistics include 200–300 cells per gene/condition and do suffer from some inter-replicate variability. A potential explanation for the *Wdr83* outlier may lay in the mechanism by which BER increases noise via DNA topology changes. Specifically, the BER enzyme AP endonuclease 1 (Apex1) generates DNA supercoiling, which leads to an accumulation of RNA polymerase II; when released, this amplifies transcriptional burst size. Indeed, the IdU noise effect can be phenocopied by topoisomerase inhibitors, which increase DNA supercoiling.^20^ Consequently, regions that naturally have increased supercoiling and corresponding high levels of topoisomerase may be less sensitive to changes in topology caused by IdU- and BER-induced supercoiling. It has been previously reported that topologically associated domain (TAD) boundaries are regions that exhibit substantial supercoiling and are enriched in insulator binding protein CTCF^45^^,^^46^ and that topoisomerase IIB is prevalent at CTCF sites.^47^ Intriguingly, *Wdr83* has two relatively unique features among the panel of genes tested: (1) it contains a relatively large CTCF binding region and (2) it is found at a TAD boundary.^45^ Together, these two features may be consistent with IdU acting as a topology-dependent global noise-enhancer molecule, as shown by scRNA-seq (Figure 1) and smFISH (Figure 2) analysis.

A second point of interest is the case of *Sox2*. While most genes analyzed exhibited a homeostatic increase in noise—without a substantial change in mean—smFISH revealed that IdU induced a decrease in mean *Sox2* mRNA, though we note that the change in mRNA numbers was less than 2-fold. Notably, IdU did not alter single-cell *Sox2* protein levels in our previous analysis.^20^ It is possible that the reported negative feedback regulation of *Sox2*^48^ acts to buffer changes at the protein level.

From the practical perspective of quantifying expression noise, this study reveals that common analyses can fail to resolve quantitative changes in intrinsic expression noise, particularly for individual genes. A number of approaches have been proposed to overcome these types of scRNA-seq limitations, including elegant solutions using mathematical modeling methods to address technical variability without compromising the quantification of biological noise,^40^ though these can be cumbersome to implement. The analyses herein indicate that it may be advisable to combine high-throughput analyses (e.g., scRNA-seq) with lower-throughput direct quantification (e.g., smFISH) to quantify changes in transcriptional noise for individual genes, as the “ground truth” may lie somewhere in between the results of each analysis. Regardless, both the scRNA-seq and smFISH data argue that IdU appears to be a global noise enhancer that could be leveraged to modulate noise without altering mean expression for the majority of genes.

### Limitations of the study

While all the scRNA-seq algorithms explored in this study indicate that IdU results in genome-wide homeostatic amplification of transcriptional noise, it is limited by a single replicate in mESCs and smaller sequencing depth in the two replicates of Jurkat cells. Additionally, although smFISH estimates support the scRNA-seq results in mESCs, this validation is restricted to a smaller set of target genes due to technical limitations, which may also constrain the evaluation of the algorithms using smFISH as a reference. Furthermore, variations observed across the replicates in the *Wdr83* gene, along with its contrary trend from the scRNA-seq estimates, raise questions on the contribution of topoisomerase-based relaxation of supercoiling upon IdU-induced repair versus the accessibility and effectiveness of IdU itself at the TAD regions, as well as the influence of the biophysical properties of the gene. Our analysis is limited to the relative estimates of burst size and frequency, inferred from the fitting of mRNA distributions to a negative binomial distribution under the assumption that mRNA stability is unaltered upon IdU treatment, due to the lack of experimentally measured half-life estimates for all the target mRNAs. Lastly, while our results indicate a global penetrance of IdU, this is based on the fast-dividing embryonic mouse stem cells and transformed human Jurkat cells. The extent of IdU penetrance and quantification of transcriptional noise in primary differentiated cell lines, which usually have relatively slower proliferation rates, shorter replication dwell times, and different chromatin landscapes and dynamics, remains to be explored for utilizing IdU as a noise-enhancer probe.

## Resource availability

### Lead contact

Further information and requests for resources and reagents should be directed to and will be fulfilled by the lead contact, Leor S. Weinberger (leor.weinberger@gmail.com).

### Materials availability

This study did not generate new unique reagents.

### Data and code availability

• Jurkat scRNA-seq raw and processed sequencing data have been deposited at GEO and are publicly available as of the date of publication. Accession numbers are listed in the key resources table. Microscopy data reported in this paper will be shared by the lead contact upon request.
• This study does not report original algorithm/software (however, custom code/scripts are available upon request).
• Any additional information required to reanalyze the data reported in this paper is available from the lead contact upon request.

## Acknowledgments

We thank Ravi Desai, Gustavo Vasen, Karla Delucas, and Daniel Lewis for technical guidance; Kathryn Claiborn for editing; and Oded Regev for useful discussions. Data for this study were acquired at the Nikon Imaging Center at UCSF using an 10.13039/100000002NIH S10 Shared Instrumentation grant (1S10OD017993-01A1). The Gladstone Genomics Core performed 10× Genomics library construction. Sequencing was performed at the UCSF CAT, supported by 10.13039/100008069UCSF PBBR, RRP IMIA, and 10.13039/100000002NIH 1S10OD028511-01 grants. This work was supported by 10.13039/100000002NIH award R37AI109593 (to L.S.W.). B.Z. acknowledges support from the EMBO fellowship (ALTF 388-2021), and X.C. acknowledges support from CIRM training grant EDUC4-12766. X.G.A. acknowledges support from 10.13039/100000002NIH training grant K12GM081266.

## Author contributions

Idea conception, X.C. and L.S.W.; conceptualization, X.C., G.P.C., N.K., B.Z., and L.S.W.; methodology, X.C., N.K., B.Z., and G.P.C.; investigation, N.K., B.Z., G.P.C., and X.G.A.; formal analysis, N.K. and B.Z.; visualization, N.K., B.Z., and G.P.C.; data curation, N.K. and B.Z.; writing – original draft, G.P.C., X.C., N.K., B.Z., and L.S.W.; writing – review & editing, N.K., B.Z., X.C., X.G.A., G.P.C., and L.S.W.; project administration, N.K. and L.S.W.; resources, funding acquisition, and supervision, L.S.W.

## Declaration of interests

L.S.W. is an equity co-founder of Autonomous Therapeutics, Inc., and Institute for Evolvable Medicines (non-profit).

## STAR★Methods

### Key resources table

**Table:** 

REAGENT or RESOURCE	SOURCE	IDENTIFIER
**Critical commercial assays**		

Chromium Next GEM Single Cell 3′ HT Kit v3.1	10x Genomics	1000370
NovaSeqX 10B flow cell (v1.5 reagents)	Illumina	20085594
10x Cell Ranger (6.1.2, 7.0.1)	10x Genomics	https://www.10xgenomics.com/support/software/cell- ranger/downloads#download-links

**Chemicals, peptides, and recombinant proteins**		

DAPI	Thermo Fisher Scientific	Cat#D1306
IdU	Millipore Sigma	Cat# I7125
Formaldehyde	Tousimis	Cat#1008A
Formamide (deionized)	Ambion	Cat#AM9342
20x SSC buffer	Invitrogen	Cat#AM9770
Glycerol	Thermo Fisher Scientific	Cat#17904
Glucose oxidase	Sigma Aldrich	Cat#G7141
Catalase	Sigma Aldrich	Cat#C3515
Imaging chamber (8-well dish)	Ibidi	Cat#80826

**Deposited data**		

Single-cell RNA-seq Jurkat	This study	GSE263194
Single-cell RNA-seq mESC	Desai et al. 2021^20^	GSE176044

**Experimental models: Cell lines**		

mESC	Desai et al. 2021^20^	–
Jurkat	American Type Culture Collection	TIB-152

**Oligonucleotides**		

smRNA-FISH probes, See Data S3	This study (Stellaris, LGC Biosearch Technologies)	N/A

**Software and algorithms**		

Fiji/ImageJ	Schneider et al. 2012^49^	https://imagej.nih.gov/ij/
MATLAB (R2024a)	MathWorks^50^	https://www.mathworks.com/products/matlab.html
R (4.4.0)	–	–
FISH-Quant (version – v2a)	Mueller et al. 2013^38^	https://bitbucket.org/muellerflorian/fish_quant/src/master/
BASiCS (version 2.15.5)	Vallejos et al. 2015,^31^ Eling et al. 2019^32^	https://www.bioconductor.org/packages/3.7/bioc/html/BASiCS.html
Linnorm (2.28.0)	Yip et al. 2017^30^	https://www.bioconductor.org/packages/release/bioc/html/Linnorm.html
SCnorm (1.26.0)	Bacher et al. 2017^33^	https://www.bioconductor.org/packages/release/bioc/html/SCnorm.html
SCTransform (0.4.1)	Hafemeister, C., and Satija, R, 2019^28^	https://cran.rproject.org/web/packages/sctransform/index.html
Scran (1.30.2)	Lun et al. 2016^29^	https://bioconductor.org/packages/release/bioc/html/scran.html

### Experimental model and study participant details

#### Cell culture

Mouse embryonic stem cells (E14, male) were cultured on gelatin-coated platers with ESGRO- 2i/LIF medium (Millipore, cat: SF002-500) at 37°C, 5% CO2, in humidified conditions, as described previously.^20^ Jurkat T Lymphocytes were cultured in RPMI-1640 medium (supplemented with L-glutamine, 10% fetal bovine serum, and 1% penicillin-streptomycin), at 37°C, 5% CO_2_, in humidified conditions. Cells were treated with either DMSO or 10/20 μM IdU (I7125, Millipore Sigma) for 24/48h in mESC/Jurkat cells respectively.

### Method details

#### Single-cell RNA sequencing analysis (mESCs and Jurkat cells)

For mESCs, we used our previously published^20^ deeply sequenced scRNA-seq dataset available at the GEO repository under accession number GEO:GSE176044. Read mapping to mm10 genome was done with CellRanger (6.1.2)^51^ to obtain a gene count matrix. Before applying each normalization method, both DMSO and IdU datasets were subjected to a quality control process. First, Seurat^52^ was used to filter for high-quality cells using a minimum of 4000 detected genes, 10000 UMI counts, and <10% reads mapping to mitochondrial genes per cell. The resulting count matrix was then further filtered by the ‘BASiCS_Filter’ function from the BASiCS^31^ R package with default parameters, which limited the analysis to genes with sufficient sequencing coverage for reliable noise quantification. The output count matrix consisted of 811 cells for DMSO and 732 cells for IdU across 4456 genes. This output was then used to run five normalization pipelines according to their protocols: SCTransform,^28^ scran,^29^ Linnorm,^30^ BASiCS,^31^ and SCnorm.^33^ For comparison, the filtered output was also normalized using a simple approach referred to as the “raw” method, here. In this method, the gene-specific counts in each cell were divided by the total counts in that cell and then scaled by a factor of 10^4^. BASiCS was run with default parameters and recommended settings (*N* = 20000, Thin = 20 and Burn = 10000) using the horizontal integration strategy (no-spikes). The normalized data obtained from ‘BASiCS_DenoisedCounts’ function was further normalized similar to the raw data. The other packages were run with default parameters.

For Jurkats, two biological replicates (4 samples) of single-cell suspensions were loaded on a Chromium X instrument using a Chromium Next GEM Single Cell 3′ HT Kit v3.1 (10x Genomics). Sequencing was performed on an Illumina NovaSeq 6000 with a paired-end setup specific for 10x libraries. Data were aligned to hg38 reference genome using 10x Cell Ranger (7.0.1). Resulting sequencing depth as estimated by median UMI count per sample is shown in Figure S3E. Analysis was performed similarly to the mESC dataset: Seurat was used to filter for high-quality homogeneous cell population using the following filters: 3000 < detected genes <5000, 8000 < UMI counts <15000, and <10% reads mapping to mitochondrial genes per cell. The resulting count matrix was then further filtered by the BASiCS_Filter function with default parameters. The output count matrix consisted of 5988 and 5786 cells for DMSO samples and 5148 and 4701 cells for IdU samples across 1107 genes. This output was then used to run the above-mentioned 5 normalization pipelines according to their protocols. For each sample, we calculated separately for each biological replicate the mean, CV^2^, and Fano values per gene and then averaged the values of both replicates for further analysis.

#### Single molecule RNA FISH

Cells within 3–12 passages were used for smRNA FISH experiments. Probes for the detection of transcripts were developed using the designer tool from Stellaris (LGC Biosearch Technologies) (Data S3) setting the minimum number of probes to 30 (TAMRA conjugated) for gene transcripts.

1.5 x10^5^ mouse embryonic stem cells were seeded into well of a gelatin-coated, 8-well Ibidi dish (cat: 80826) in 2i/LIF media. 24 h following seeding, media was replaced with 2i/LIF containing 10 mM IdU or equivalent volume DMSO. After 24 h of treatment, cells were then fixed with DPBS in 4% paraformaldehyde for 10 min. Fixed cells were washed with DPBS and stored in 70% EtOH at 4°C for 1 h to permeabilize the cell membranes. Probes were diluted 200-fold and allowed to hybridize at 37°C overnight. Wash steps and DAPI (Thermo, cat: D1306) Wash steps and DAPI (Thermo, cat: D1306) staining were performed as described (https://www.biosearchtech.com/support/resources/stellaris-protocols). Briefly, after 16 h, cells were washed with wash buffer and incubated for 30 min at 37°C twice, followed by DAPI stain (DAPI in wash buffer at 10 μg/ml), for 15 min at 37°C. The cells were washed with 2x SSC (Invitrogen, cat: AM9770) once, followed by freshly prepared GLOX solution and incubated for 3 min. The cells were finally suspended in the anti-fade GLOX buffer with enzymes (i.e., also the imaging buffer) to minimize photo-bleaching (buffer containing 50% glycerol (Thermo, cat: 17904), 75 mg/mL glucose oxidase (Sigma Aldrich, cat: G7141), and 520 mg/mL catalase (Sigma Aldrich, cat: C3515). Images were collected on an inverted Nikon TiE microscope (Nikon) run using Micromanager 2.0^53^ equipped with a CSU-W1 Spinning Disk with Borealis Upgrade (Yokogawa, Andor), ILE Laser launch with 4 laser lines (450/488/561/646nm, Andor), quad-band dichroic ZT405/488/561/647 (Chroma), emission filters for DAPI (ET447/60), GFP (ET525/50), RFP (ET607/36), and Cy5 (ET685/40) (Chroma), piezo XYZ stage (ASI), and Zyla 4.2 CMOS camera (Andor), using a Plan Apo VC 60x/1.4 Oil objective (Nikon). Approximately 10 XY regions of interest were randomly selected for each condition. For each image, XY pixel size was 108nm/px, and a Z-step size of 250nm was used with over 60 image planes to fully cover the tissue. The additional replicates were imaged on a confocal laser scanning microscope (Fluoview 3000 Olympus) with a 63x (1.4 NA) oil-immersion objective. Approximately, 10-15 XY regions were randomly selected for each condition at z-step size of 280nm for about 40–60 frames to span the cells. Prior to imaging, cells were checked for Nanog-GFP presence to confirm the pluripotent state of the mESCs.

#### Image analysis and extrinsic noise filtering for quantification

Image analysis and spot counting was performed using FISH-quant.^38^ Images, were background subtracted with a rolling ball radius of 10 pixel in the TAMRA channel for the control and treated sets requiring pre-processing in Fiji.^49^ Cells were manually segmented to ensure selected cells for the analysis were: (i) single and non-overlapping in all dimensions; (ii) non-dividing (based on cell shape) and those at the later stages of cell-division (such as metaphase and anaphase) were excluded based on DAPI staining; and (iii) of similar size to minimize extrinsic noise. To remove outliers, for every pair of probes per replicate, cells with areas below 5^th^ and above 95^th^ percentile (calculated from the combined DMSO and IdU population) were excluded. This was followed by iteratively eliminating cells with 2 and 98 percentile thresholds until the area distributions of DMSO and IdU satisfied statistical insignificance (Permutation test, a = 0.01, number of permutations with replacement = 10000) and the correlation coefficient for the linear relationship between mRNA abundance and cell-size was less than <0.45 (24/28 cases had *p* > 0.05 and R^2^ < 0.3). This approach does not assume a prior-distribution and removes any bias that could stem from differences in the number of cells per group. For, all the analysis, gene-sets with at least >50 cells/treatment were considered, (except for one *Farsa* and *Syntaxin7* replicate with ∼25 and ∼35 cells/condition). The lack of cell-size dependent effects was further confirmed by computing the cell-size corrected noise-metric as described,^39^ which yielded no changes in the measured metrics per gene per replicate. (a total of 2201 DMSO and 2035 IdU treated cells were analyzed after extrinsic filtering from a total of 2748 and 2693 segmented cells.).

#### Estimation of burst size and burst frequency

The distribution of mRNA/cell for each gene and replicate were fit to the negative binomial distribution using maximum likelihood estimates (nbinfit, MATLAB). The estimated burst size and burst frequency from the fits were inferred as: mean = burst size x burst frequency and Fano factor is 1 + burst size (Figures S6A and S6C). Data from all replicates was also pooled for each gene and the mRNA distributions fit to estimate the bursting-parameters.

### Quantification and statistical analysis

Statistical analysis was performed in MATLAB. To test for the homeostatic noise amplification, increase in transcriptional noise between DMSO and IdU treated samples in smFISH data, the mean of the measures over the replicates were considered. A non-parametric paired Wilcoxon signed rank was used to test for significance in mean (two-sided). While a one-sided paired Wilcoxon signed rank test was used to test for the significance for increase in noise and burst size, and decrease in burst-frequency (based on the mechanism of IdU shown previously^20^ and from the scRNA-seq analysis in this study). The Permutation test was used to filter extrinsic noise and test for the significance in cell-area distributions between DMSO and IdU cells in smFISH data. To evaluate the performance of the scRNA-seq methods for homeostatic noise amplification, a combined score (S) was computed for each method (k); defined as the linear sum of the medians ‘χ′, computed for each metric (i.e., *‘i’*). The metrics ($\chi_{i}^{k}$) are relative deviations in mean expression (*μ*), squared coefficient of variation ($\frac{\sigma 2}{\mu^{2}}$) and Fano factor ($\frac{\sigma 2}{\mu }$) from scRNAseq method ($D_{j}^{k}$) and smFISH (*E*_*j*_) across all the genes (*‘j’*). The minimal score *S*_*k*_ corresponds to the method that matches the smFISH closely.

$$S_{k}=\sum_{i\in \left\{\mu ,\frac{\sigma^{2}}{\mu^{2}},\frac{\sigma^{2}}{\mu }\right\}}\chi_{i}^{k}$$

$$\chi_{i}^{k}=median{\left\{{\left(\frac{D_{j}^{k}-E_{j}}{E_{j}}\right)}^{2}\right\}}_{j=1:n}$$

## Supplemental information

Document S1. Figures S1–S6

Data S1. Processed data: mESCs Statistics of gene expression counts normalized by the indicated method in the “method” column. Columns contain mean, CV^2^ and Fano values for DMSO and IdU samples, as well as the log2 fold-changes (IdU/DMSO) for each of the statistics. “diff_Fano_2” and “diff_Fano_bin” contain fold-changes (not log2) used for Figure 1D.

Data S2. Processed data: Jurkat, related to Figure S2D As Data S1, for Jurkat dataset.

Data S3. smFISH probe list Sequences of smRNA-FISH oligonucleotide probe for eight genes.

Document S2. Article plus supplemental information
